# Alterations in the axon initial segment plasticity is involved in early pathogenesis in Alzheimer's disease

**DOI:** 10.1002/mco2.768

**Published:** 2024-10-14

**Authors:** Yu Li, Han Wang, Yiming Wang, Zhiya Chen, Yiqiong Liu, Wu Tian, Xinrui Kang, Abolghasem Pashang, Don Kulasiri, Xiaoli Yang, Hung Wing Li, Yan Zhang

**Affiliations:** ^1^ State Key Laboratory of Membrane Biology School of Life Sciences Peking University Beijing China; ^2^ Centre for Advanced Computational Solutions (C‐fACS) AGLS Faculty Lincoln University Canterbury New Zealand; ^3^ Division of Life Sciences and Medicine Department of Neurology Institute on Aging and Brain Disorders The First Affiliated Hospital of USTC University of Science and Technology of China Hefei China; ^4^ Neurodegenerative Disorder Research Center Anhui Province Key Laboratory of Biomedical Aging Research Division of Life Sciences and Medicine University of Science and Technology of China Hefei China; ^5^ Department of Chemistry The Chinese University of Hong Kong Hong Kong China

**Keywords:** Alzheimer's disease, ankyrin G, axon initial segment, plasticity

## Abstract

Alzheimer's disease (AD) is the most prevalent neurodegenerative disorder, characterized by the early presence of amyloid‐β (Aβ) and hyperphosphorylated tau. Identifying the neuropathological changes preceding cognitive decline is crucial for early intervention. Axon initial segment (AIS) maintains the orderly structure of the axon and is responsible for initiating action potentials (APs). To investigate the role of AIS in early stages of AD pathogenesis, we focused on alterations in the AIS of neurons from APP/PS1 mouse models harboring familial AD mutations. AIS length and electrophysiological properties were assessed in neurons using immunostaining and patch‐clamp techniques. The expression and function of ankyrin G (AnkG) isoforms were evaluated by western blot and rescue experiments. We observed a significant shortening of AIS in APP/PS1 mice, which correlated with impaired action potential propagation. Furthermore, a decrease in the 480 kDa isoform of AnkG was observed. Rescue of this isoform restored AIS plasticity and improved long‐term potentiation in APP/PS1 neurons. Our study implicates AIS plasticity alterations and AnkG dysregulation as early events in AD. The restoration of AIS integrity by the 480 kDa AnkG isoform presents a potential therapeutic strategy for AD, underscoring the importance of targeting AIS stability in neurodegenerative diseases.

## Graphical abstract

• The length of the AIS is reduced in neurons from AD patients or animal models, which slows down the velocity of action potential propagation on the axon.

• The structural plasticity of the AIS is damaged in neurons from APP/PS1 mice.

• Ankyrin G (AnkG), the key organizer of the AIS, is down‐regulated in APP/PS1 mice, especially the 480 kD isoform. The overexpression of 480 kD AnkG could restore the plasticity of AIS and normal LTP in AnkG^−/−^ mice.

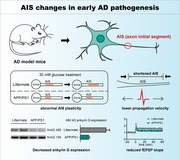

## 1 INTRODUCTION

Alzheimer's disease (AD) is the most common type of dementia in elderly.^1^ Major pathological hallmarks in the central nervous system (CNS) of AD include deposition of amyloid β (Aβ) extracellularly, formation of neurofibrillary tangles intracellularly, and dramatic loss of neuronal cells, indicating reduction of brain volume in late stage of the disease.^2^, ^3^ Noticing that decades before cognitive decline, AD patients begin to show remarkably increased level of Aβ and tau in their cerebrospinal fluid (CSF) and plasma.^4^ It is important to identify the earliest pathological changes during AD development. In the current study, we aimed to search for early alterations of neurons. Although only accounted for small proportion of AD patients, patients carrying familial AD (FAD) mutations contributes significantly to our understanding of AD pathogenesis.^5^ Animal models harboring these human‐derived FAD mutations help to elucidate the mechanisms of AD development.^6^, ^7^, ^8^ Among these causative mutations of AD, Swedish mutation of amyloid precursor protein (APP) and deletion of exon 9 of presenilin 1 (PS1ΔE9) are related to enhance Aβ production or reduce its clearance.^9^, ^10^ Previous research has showed differences between AD model mouse neurons and wild‐type mouse neurons. Whole‐cell voltage clamp recording showed an increase frequency of spontaneous excitatory postsynaptic currents in different months of age in *App* ^NL‐G‐F^ mice.^11^ Also, an immunostaining result with structured illumination microscopy (SIM) in the initial part of the axon showed that the periodicity of the axon was disrupted in cultured neurons from APPswe/PS1ΔE9 (APP/PS1 in short) mice.^12^

In the axon, the axon initial segment (AIS) is one of the most vulnerable sites for injury and damage because of its complicated structure and function.^13^, ^14^, ^15^ The AIS is a highly structured region with scaffolding proteins, such as microtubules and actin, adaptor proteins, such as ankyrin G (AnkG) and βIV‐spectrin, transmembrane cell adhesion molecules, such as neurofascin 186, and a dense population of ion channels.^16^, ^17^ The AIS plays two main roles in neurons. One is as the initiating point of action potential (AP) on the axon; another function is responsible for maintaining the polarity of neurons, so that the organization of the axon is significantly different from the soma and dendritic area.^18^, ^19^ The AIS is composed of membrane proteins, dense submembrane structures, and cytoskeletons.^18^, ^20^ The AnkG protein acts as an anchoring protein to bind to membrane proteins with submembrane proteins and cytoskeleton together.^14^, ^21^ Abnormalities in AnkG can cause the loss of AIS, decrease of the number of nodes of Ranvier,^22^ and disturbance on GABAergic synaptic transmission.^23^ Previous studies of Dr. Bennett and his colleagues showed that the giant AnkG (480 kDa) recruits the βIV‐spectrin and ion channels, which ensures the normal formation of the AIS.^22^, ^24^

Evidence has suggested that the AIS can undergo modulation during both physiological and pathological conditions. The length of the AIS can vary as a result of receiving a series of stimuli or deprivation of stimuli.^25^ Input from sensory system can cause rapid changes in the AIS, and this change induces alterations in its excitability.^26^ Besides, AIS in disease conditions also undergo plastic changes. Deprivation of auditory input made the AIS in the avian cochlear nucleus prolonged.^27^ It was also reported that human‐induced pluripotent stem cells (hiPSCs)‐derived motor neurons from amyotrophic lateral sclerosis (ALS) background showed prolonged length of AIS and impaired plasticity in the AIS.^28^

In this study, we found that the length of AIS in neurons from AD model mice was shortened. Alterations of AIS length‐related electrophysiological characteristics were described in modeling and confirmed in APP/PS1 neurons in vitro using membrane potential probes. Our study identified the abnormal plasticity of the AIS in APP/PS1 mouse model of AD and confirmed that this change was associated with the reduction of AnkG. We further identified the association between abnormalities in AIS plasticity and alterations in long‐term potentiation (LTP), using a defective expression line of AnkG.

## 2 RESULTS

### 2.1 The AIS of neurons from AD patients and AD model mice were shortened

**FIGURE 1:**
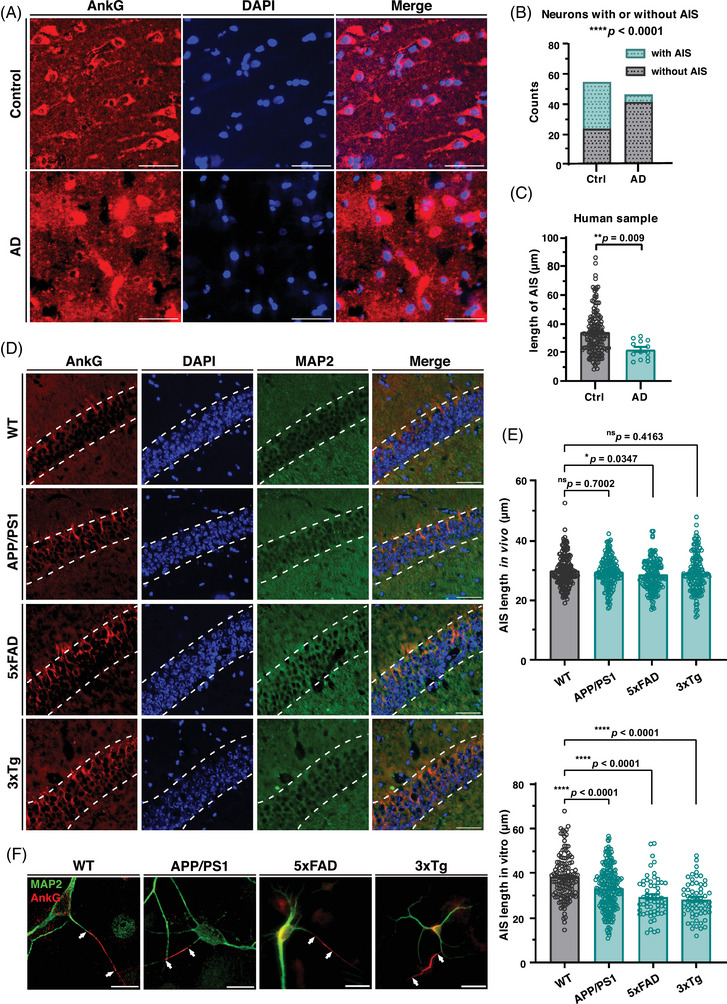
Neurons in vivo and in vitro from Alzheimer's disease (AD) model mice and human brain samples have altered axon initial segment (AIS) lengths. **(A)** Immunofluorescence staining of paraffin‐sectioned samples of human cerebral cortex. Red, AnkG (AIS); blue, DAPI (nuclei). Scale bar, 50 µm. (**B)** Statistics of staining results in (**A)**. The number of neurons clearly visible with or without AIS was counted in the two samples. Data analyzed by Fisher's exact test. (**C)** Statistics performed on the lengths of the AIS in brain slices from human samples. Student's *t*‐test was used for analysis. (**D)** Immunofluorescence staining in coronal frozen sections at the CA1 region of the hippocampus. Slices were from 6‐month‐old wild‐type, APP/PS1, 5xFAD, and 3xTg mice. The dashed line is the outline of the cluster of cell bodies in the CA1 region of the hippocampus. Red, AnkG (AIS), green, MAP2 (soma and dendrites), blue, DAPI (nuclei). Scale bar, 50 µm. (**F)** Immunofluorescence staining of DIV 7 cultured neurons from wild‐type, APP/PS1, 5xFAD, and 3xTg E18.5 mice. The segment between the two arrow marks is the AIS identified by AnkG. Red, AnkG, green, MAP2. Scale bar, 20 µm. (**E** and **G)** Statistics performed on the lengths of the AIS in brain slices and cultured neurons from the four strains. The upper panel shows the results of brain slices, and the lower panel shows the results of cultured neurons. One‐way analysis of variance (ANOVA) was used for analysis. ns, not significant; *p < 0.05; ****p < 0.0001.

The AIS is important for maintaining neuron polarity and organization in an axon.^29^ To investigate whether changes in AIS occur during the early development of AD, we measured the AIS length of neurons in vitro and in vivo from three kinds of AD model mice: APPswe/PS1dE9 (APP/PS1 in short), 5xFAD, and 3xTg. The onset of cognitive impairment and Aβ deposition differ among these three strains, with 5xFAD strain showing cognitive impairment at an early age (4–5 months), and Aβ deposition occurring as early as 2 months. APP/PS1 strain shows cognitive impairment relatively later (at 10‐12 months), and the 3xTg is the only one with tau‐related mutations, with cognitive impairment appearing around 3−6 months, and Aβ deposition occurring after 9 months of age.^6^, ^8^ We are more interested in the early changes before cognitive impairment, so we choose the age of 6 months. At this time point, APP/PS1 model has just begun to show amyloid plaque deposition, but cognitive impairment has not yet occurred, representing the early stage of AD. The other two strains, however, have already showed changes in cognitive abilities at 6 months of age, thus serving as subjects for the study of the disease phase and as a control for relatively early changes. The immunostaining results of brain slices showed that the length of the AIS in hippocampal CA1 neurons of 6‐month‐old 5xFAD mice was significantly shorter than that of C57BL/6 wild‐type control mice, while the other two mild strains APP/PS1 and 3xTg showed no significant change (Figure 1D,E).

In order to detect the earliest possible changes in neurons in vitro, we selected neurons that had already reached a basic complete morphology but their axons had not yet fully developed, specifically neurons at 7 days in vitro (7 DIV), as the subjects for our experiments. However, in vitro cultured neurons were measured, fixed, and measured at 7 DIV, and significant AIS length shortening occurred in all three AD model mice (Figure 1F,G). This phenomenon was confirmed indirectly in human brain slice samples (Figure 1A), where the length of AIS was significantly shortened in brain slices from AD patients and, notably, the frequency of clearly visible AIS was greatly reduced in human samples (Figure 1B,C). These results suggest that the structure of the AIS was damaged during AD progression and that these deficits persist until late stages.

### 2.2 Voltage sensor QuasAr2 and modeling revealed the details of action potential changes in the AIS of APP/PS1 neurons

**FIGURE 2:**
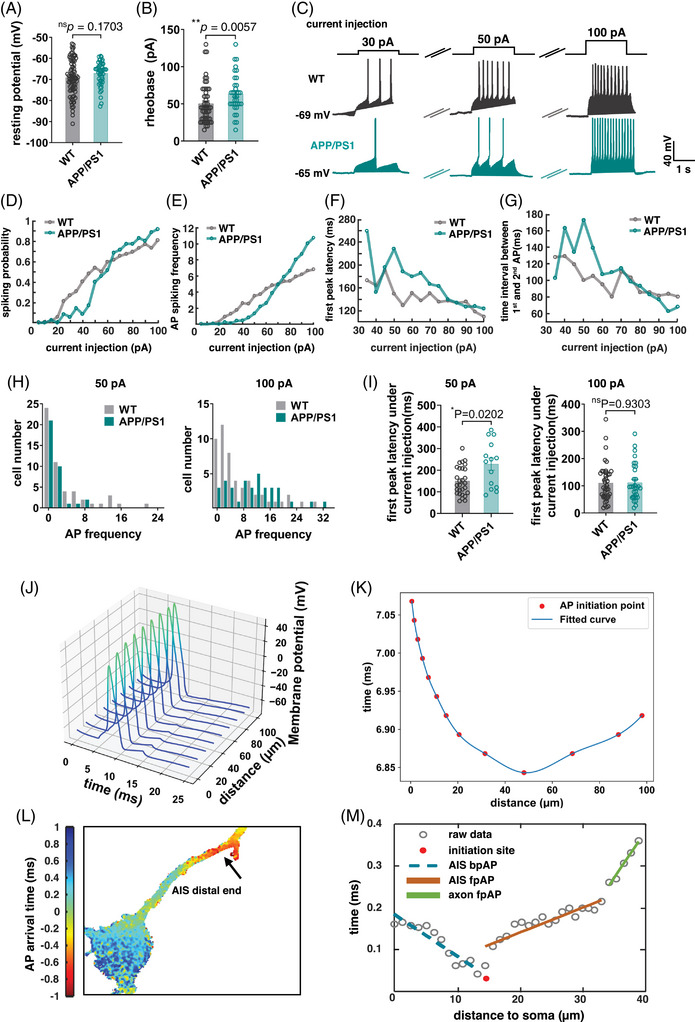
The electrophysiological characteristics of APP/PS1‐cultured neurons are altered in vitro. (**A)** Whole‐cell patch‐clamp experiments were performed to record the resting potentials of cultured neurons at DIV 7 from WT or APP/PS1 E18.5 mice. The Student's *t*‐test was used for statistics. (**B)** Whole‐cell patch clamp was used to test rheobase. The minimum injected current that could generate an action potential in neurons from APP/PS1 or wild‐type mice is obtained by multi‐stimuli. (**C)** An example of the cell membrane potential detected using different injected currents. (**D)** Statistics of action potential spiking probability under different injection currents. (**E)** Statistics of action potential (AP) spiking frequency under different injection currents. (**F)** Statistics of first peak latency under different injection currents. (**G)** Statistics of time interval between the first two APs. (**H)**. AP frequency distribution of neurons from wild‐type or APP/PS1 mice at an injection current of 50 pA (left) and 100 pA (right). (**I)** Statistics of first peak latency of neurons from wild‐type or APP/PS1 mice at an injection current of 50 pA (left) and 100 pA (right). (**J)** AP curves at different locations in the axon initial segment (AIS) simulated using a static model. (**K)** Timing of action potential bursts at different locations on the AIS in the computer simulation model. (**L)** Temporal and spatial distribution of membrane potential changes following an action potential burst. Different colors represent different lengths of times between membrane potential changes and action potential bursts. The positive time represents signal conduction to the distal end of the axon, and the negative time represents reversal conduction to the soma. (**M)** Scatter plot of the relationship between the time the voltage sensor displays fluorescence and the relative location of the fluorescence display after the action potential is spiking.

To clarify the association between changes in the AIS and the development of AD pathology, we analyzed the electrophysiological signals of neurons in APP/PS1 mice (Figure 2). Whole‐cell patch‐clamp results for APP/PS1 showed that resting potential was not significantly different from that of the control (Figure 2A), while rheobase was significantly higher than that of the control (Figure 2B). Using different sizes of injected membrane currents for stimulation (Figure 2C), APP/PS1 mice were found to be more excited at high currents and less excitatory at low currents (Figure 2D–I). These results suggested changes in electrophysiological characteristics of neurons cultured in vitro from APP/PS1 mice. AIS is the starting point of AP on the axon of neurons. It can be simulated by modeling and analysis using NEURON (Figure 2J), and AP on the AIS starts in the middle position and spreads to both ends (Figure 2K). The simulation results indicated that a shorter AIS led to a decrease in the propagation velocity of APs. To understand the pathological changes in AIS during AP generation, voltage sensor QuasAr2 described previously^30^ was used to analyze the process of AP generation in the AIS (Figure 2L).

**FIGURE 3:**
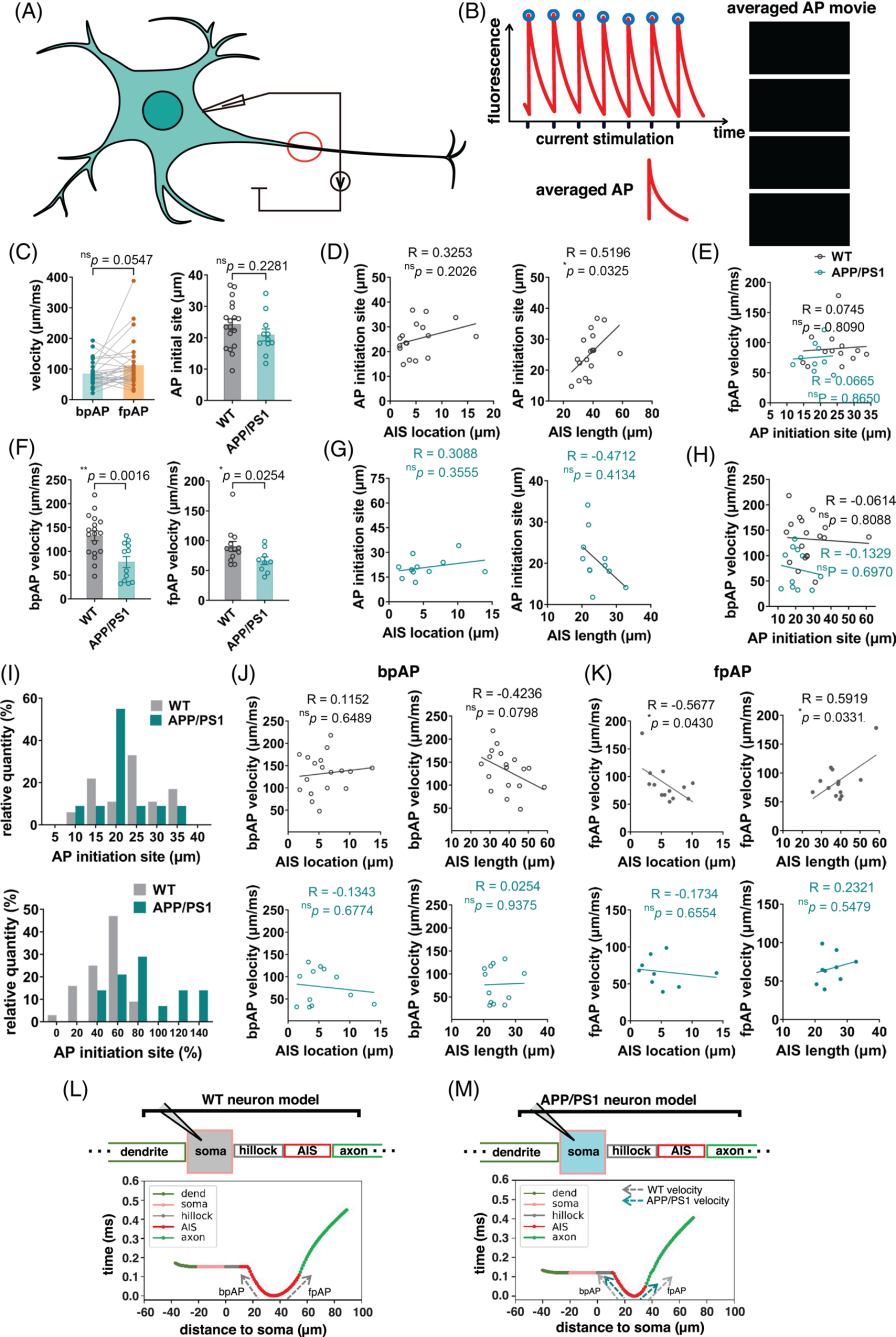
**Abnormal AP propagation of APP/PS1 neurons at** axon initial segment (the **AIS) was detected by membrane potential probe. (A)** Schematic of fluorescence imaging of cells stimulated with single‐cell patch clamp. Cells were transfected with QuasAR2, a membrane potential probe capable of giving a fluorescent signal in response to membrane potential changes at one day before imaging. Cultured neurons in days in vitro (DIV) 7 were stimulated with patch clamp to generate action potentials, and the stimulation was accompanied by observation of neuronal axonal fluorescence signals. Neurons were fixed and the AIS were labeled by AnkG after recording. (**B)** Schematic of real‐time membrane potential imaging of neurons initiating action potentials. Action potentials were fired periodically and the fluorescence intensity at different locations of the neurons was observed simultaneously. Imaging was taken with interval of 2 ms. (**C)** (Left) Propagation velocities of bpAP and fpAP generated at the AIS calculated by linear fitting in wild‐type neurons. (Right) Location of action potential initiation in APP/PS1 mouse neurons versus wild‐type neurons. (**F)** (Left) Propagation velocity of AIS‐initiated bpAP in APP/PS1 mouse neurons versus wild‐type neurons. (Right) Propagation velocity of AIS‐initiated fpAP in APP/PS1 mouse neurons vs. wild‐type neurons. (**D** and **G)** Relationship of the AIS position or length of a neuron to the action potential (AP) initiation site. (**D)** Wild‐type neurons (black) and (**G)** APP/PS1 neurons (dark cyan). The two left panels show the relationship between AIS position and AP start while the two right panels show the relationship between AIS length and AP position. All data were analyzed by linear regression fitting. (**E** and **H)** Relationship between neuronal fpAP (**E**) or bpAP (**G**) and AP initiation. Data were analyzed by linear regression fitting. (**I)** Absolute (top) and relative (bottom) location distributions of AP initiation locations in APP/PS1 and wild‐type neurons. (**J)** Relationship of the AIS position or length of a neuron to bpAP velocity. The top two panels show wild‐type neurons (black) and the bottom show APP/PS1 neurons (dark cyan). The two left panels show the relationship between AIS position and AP start, while the two right panels show the relationship between AIS length and AP position. All data were analyzed by linear regression fitting. (**K)** Relationship of the AIS position or length of a neuron to fpAP velocity. The top two panels show wild‐type neurons (black) and the bottom show APP/PS1 neurons (dark cyan). The two left panels show the relationship between AIS position and AP start, while the two right panels show the relationship between AIS length and AP position. All data were analyzed by linear regression fitting. (**L** and **M)** The spatial‐temporal information of membrane potential probe data was integrated and fitted to show the spatial‐temporal characteristics of action potential bursts on AIS. (**L**) Data obtained from WT neurons. (**M**) Data obtained from APP/PS1 neurons. The shortened length of the AIS of APP/PS1 contributes to its altered action potential signature.

After the voltage sensor was transfected into the neurons, patch‐clamp stimulation was used to induce fluorescence changes under a high‐speed fluorescence microscope (Figure 3A), and the results were fitted after multiple filming (Figures 2M and 3B and Figure S1). The results indicated that AP was generated in the middle of the AIS and transmitted to both sides, consistent with the model predictions. Comparison of APP/PS1 neurons and wild‐type neurons revealed that AP propagation was slower and less correlated with AIS length in APP/PS1 neurons (Figure 3C,F). APs propagated simultaneously toward both the distal axon and soma, generating back‐propagation AP (bpAP) and forward‐propagation AP (fpAP), respectively (Figure 3C–H). The velocity of the bpAP on the AIS was not significantly different from that of the fpAP (Figure 3C). The fpAP velocity of wild‐type neurons was associated with AIS location and length, whereas this association was lost in APP/PS1 neurons (Figure 3D‐H,J–K). Another finding consistent with the model's prediction was that the location of AIS AP onset was dependent on absolute rather than relative position on the AIS (Figure 3I). The model analysis showed that the difference in fpAP velocity between the two different neurons was related to the reduced AIS length (Figure 3L‐M).

### 2.3 AIS plasticity was disrupted in neurons from APP/PS1 mice

**FIGURE 4:**
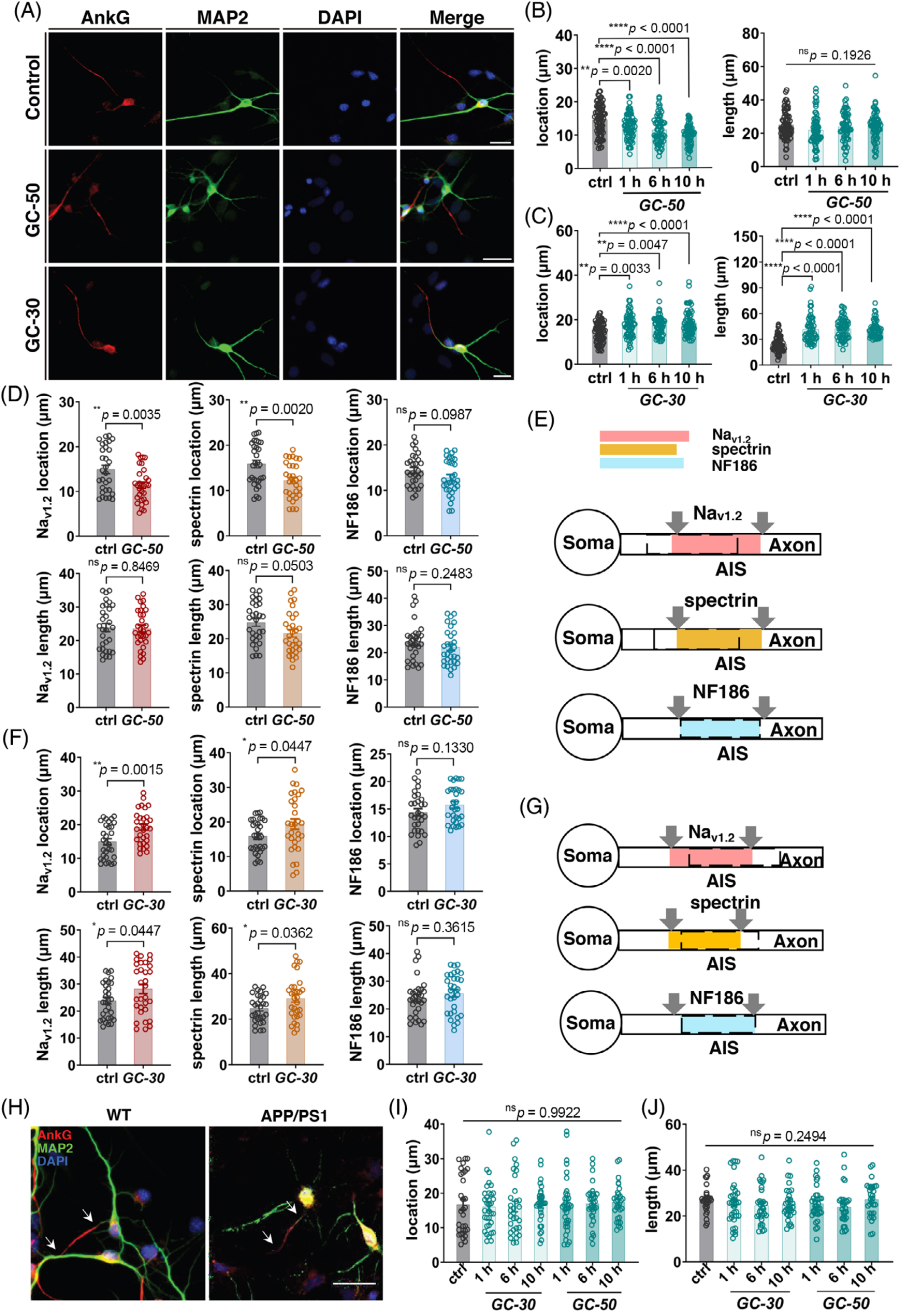
Axon initial segment (**AIS) plasticity could not be induced in APP/PS1 neurons *in vitro*. (A)** The length of AIS and the distance to the soma can be changed by using a certain concentration of glucose. DIV 7 neurons were treated with 30 mM or 50 mM D‐glucose for 10 h, then fixed and stained by AnkG antibody. GC‐30 or GC‐50 stands for being treated with 30 mM or 50 mM D‐glucose. Red, AnkG; green, MAP2; blue, DAPI. Scale bar, 20 µm. (**B)** Statistics of the location and length of AIS AnkG recorded after neurons were treated with 50 mM glucose for 1, 6, or 10 h. (**C)** Statistics of the location and length of AIS AnkG recorded after neurons were treated with 30 mM glucose for 1, 6, or 10 h. The Student's *t*‐test was used for statistics. (**D)** Statistics of the location and length of three AIS proteins after being treated with 50 mM glucose for 10 h. Left, sodium ion channel 1.2 (Na_v1.2_); middle, beta IV spectrin (βIV‐spectrin); right, neurofascin 186 (NF186). (**E)** A schematic diagram of the 50 mM glucose treatment described. The solid color blocks stand for the original location and length of the three proteins on the AIS. The dashed lines stand for the location and length after the glucose treatment of the corresponding proteins. (**F)** Statistics of the location and length of three AIS proteins after being treated with 30 mM glucose for 10 h. Left, sodium ion channel 1.2 (Na_v1.2_); middle, beta IV spectrin (βIV‐spectrin); right, neurofascin 186 (NF186). (**G)** A schematic diagram of the 30 mM glucose treatment described. The solid color blocks stand for the original location and length of the three proteins on the AIS. The dashed lines stand for the location and length after the glucose treatment of the corresponding proteins. (**H)** DIV7 APP/PS1 and WT neurons were treated with 50 mM D‐glucose for 10 h, then fixed and stained with AnkG antibody. White arrows indicate the ends of the AIS marked by AnkG staining. Scale bar, 20 µm. (**I)** Statistics of the location of AIS AnkG recorded after neurons from APP/PS1 E18.5 mice were treated with 30 mM glucose for 1, 6, or 10 h. (**J)** Statistics of the length of AIS AnkG recorded after neurons from APP/PS1 E18.5 mice were treated with 30 mM glucose for 1, 6, or 10 h. One‐way analysis of variance (ANOVA) was used for analysis. ns, not significant.

Previous studies reported that the brain glucose metabolism disorder significantly influence the steady state and function of neurons,^31^ and AIS length was shortened in type II diabetes mice, which suggested that there may be a correlation between glucose and AIS plasticity.^32^ To explore whether glucose concentrations could induce AIS plasticity, cultured hippocampal neurons on 7 DIV were treated with 30 mM or 50 mM D‐glucose. When considering AnkG localization as a maker for the AIS, relative higher concentrations of glucose (50 mM) results in closer location of the AIS to the soma, while not significantly altering its length. Conversely, relative lower concentrations of glucose treatment (30 mM) led to an increased distance between the AIS and soma, accompanied by elongation of the AIS (Figure 4A‐C). Additionally, we investigated three other specific proteins localized at the AIS: voltage‐gated sodium channel I type 2 (Na_v_1.2), βIV‐spectrin, and NF186. The results demonstrated that under high glucose treatment conditions, Na_v_1.2 exhibited a similar trend to AnkG regarding localization by moving closer to the soma without significant changes in length (Figure 4D). Regarding βIV‐spectrin, the alterations induced by high glucose treatment exhibited a similar pattern as observed for the above mentioned two proteins (Figure 4D). However, for NF186 under high glucose conditions, there was a notable deviation as neither its length nor position demonstrated significant modifications (Figure 4D), and the low glucose treatment group exhibited similar trends in Na_v_1.2 localization and distance from the soma, consistent with AnkG distribution (Figure 4F). Similarly, βIV‐spectrin displayed increased distance from the soma and elongated length under low glucose conditions (Figure 4F). Conversely, there was no significant alteration observed in NF186 status following low glucose treatment (Figure 4F). In summary, both Na_v_1.2 and spectrin demonstrated alterations corresponding to AnkG distribution, while NF186 remained unaffected by the experimental conditions (Figure 4E,G). We then take this series of changes as a pattern and use it to determine whether neurons can exhibit behaviors that are consistent with their normal plasticity characteristics. Considering the various changes of the AIS in APP/PS1, in vitro neurons were treated with glucose in an attempt to induce plasticity. Results showed that the cultured neurons of APP/PS1 did not develop AIS plasticity in glucose treatment, both in low and high concentrations (Figure 4H–J).

### 2.4 Key organizer protein AnkG was downregulated in APP/PS1

**FIGURE 5:**
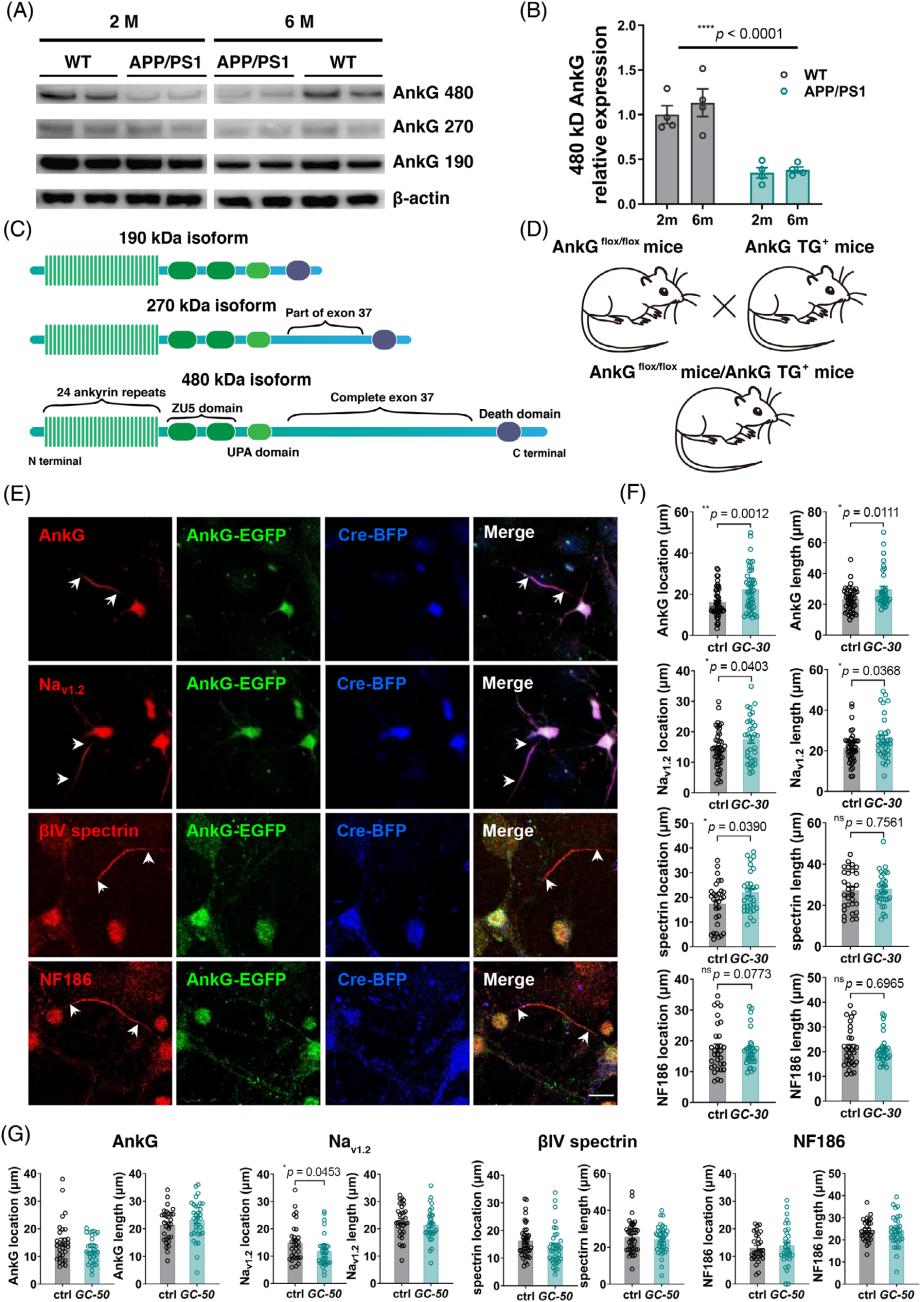
**The giant AnkG expression was decreased in the APP/PS1 hippocampus, which can restore** axon initial segment (**AIS) plasticity induced by glucose treatment. (A)** Western blot of AnkG of hippocampus from APP/PS1 mice. AnkG expression was down‐regulated in the hippocampus of APP/PS1 at 2 and 6 months of age. The hippocampus of 2‐month‐old and 6‐month‐old APP/PS1 mice and control mice were dissected out, and proteins were extracted and loaded for electrophoresis. (**B)** Statistics of the relative density of 480 kDa AnkG in western blot results described in (A). Data were normalized and analyzed by two‐way analysis of variance (ANOVA. (**C)** A schematic diagram of three main isoforms of AnkG. Only 480 kDa AnkG has full giant exon (exon 37). (**D)** A schematic diagram of how the AnkG single isoform expressing mice were produced. AnkG cKO mice and AnkG single isoform overexpressed mice were crossed. And the offspring were mated until homozygous for AnkG cKO gene. (**E)** Neurons from 480 kDa AnkG isoform specific mice (A480) were treated with glucose on DIV 3. And neurons were fixed and stained on DIV 7. (**F)** Statistics of the location and length of the four AIS proteins from A480 neurons after being treated with 30 mM glucose for 10 h. (**G)** Statistics of the location and length of the four AIS proteins from A480 neurons after being treated with 30 mM glucose for 10 h. (F and G) Data were analyzed by Student's *t*‐test.

Knowing the deficits of AIS in AD, we wanted to examine AnkG expression in the brain of AD models. There are three major isoforms of AnkG, 480 kDa, 270 kDa and 190 kDa, according to their molecular weights (Figure 5C). They differ in their functions at the AIS.^22^ Western blot results showed that 480 kDa AnkG expression was indeed decreased (Figure 5A,B). It is worth noting that this decrease occurred even when no pathological features were present (2 months of age). This implies that AnkG is already involved in the course of early AD progress. We constructed the single isoform AnkG expression lines based on the AnkG Flox mice^33^ (Figure 5D, Figure S4A). Each line could only express one isoform of AnkG in the presence of Cre (Figure S2A–C), Based on the isoform it expresses, its name is abbreviated in the form of A480 (or A270, A190). Only A190 mice showed worse performance in the behavioral tests (Figure S2D–K). These mice were also used to culture neurons in vitro. Dendritic spines could be divided into three categories by their shapes (Figure S3A and B). The number of spines per 10 µm dendrite had significant differences between A270 or A190 and their control group, while no difference was showed between A480 and the control group (Figure S3C–E). Furthermore, we induced AIS plasticity with glucose treatment in A480‐ and A270‐cultured neurons, and the results showed that the 480 kDa but not 270 kDa isoform could rescue the deficits of plasticity in the AIS, which maintained the characteristics of AIS under glucose treatment (Figure 5E–G and Figure S5). A190 mice were not included because previous studies showed that 190 kDa AnkG was not expressed specifically on the AIS.^22^ This suggested that the 480 kDa AnkG could restore the plasticity damage caused by partial loss in exon 37 of AnkG on the AIS. Overexpression of 480 kDa isoform AnkG in neurons led to no alterations in location and length of all three AIS located proteins, except Na_v1.2_ location (Figure S4B–F).

### 2.5 The attenuated LTP of APP/PS1 was associated with its impaired plasticity in the AIS

**FIGURE 6:**
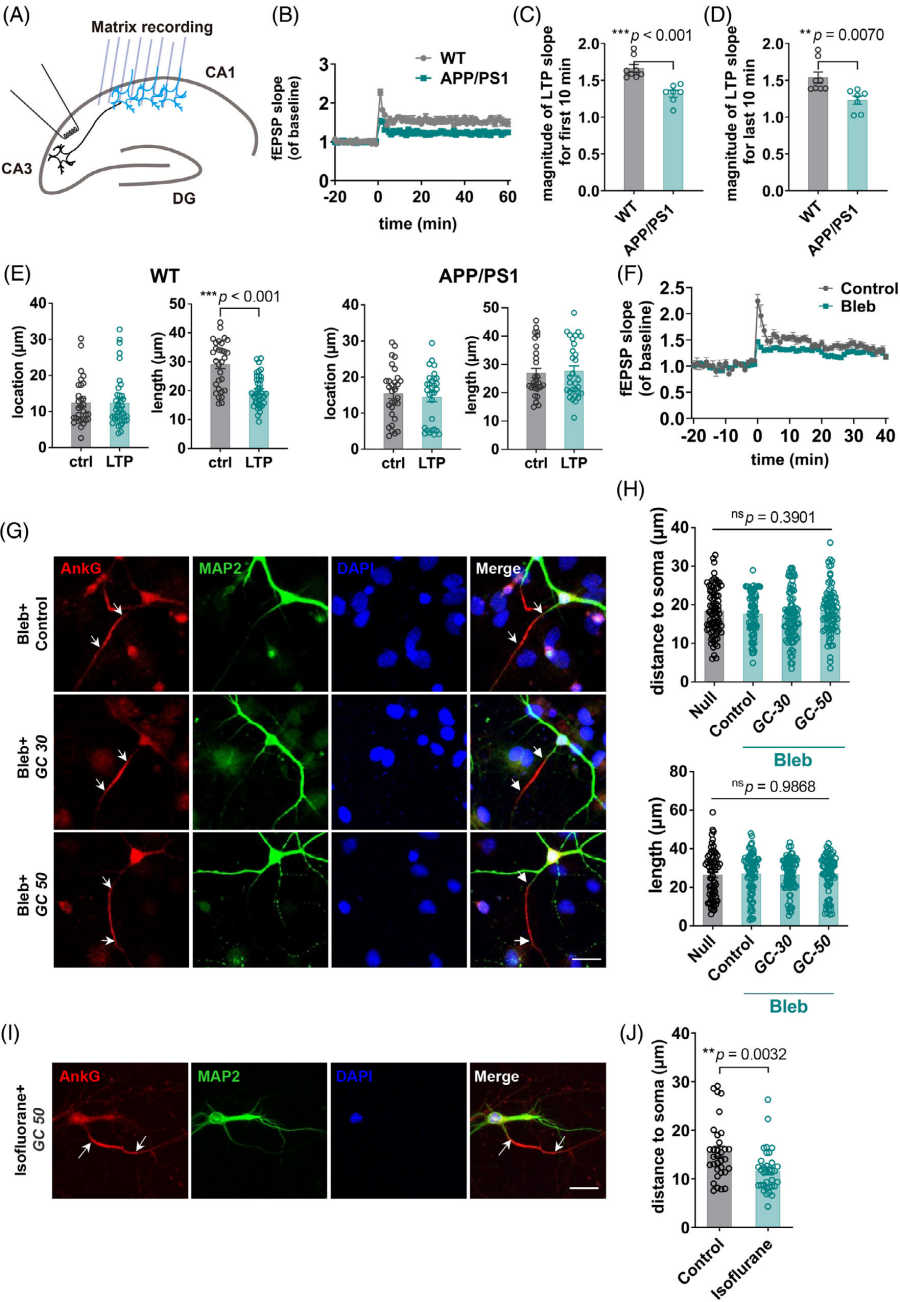
**Inhibition of** axon initial segment (**AIS) plasticity resulted in failure to properly induce** long‐term potentiation (**LTP) generation. (A)** Schematic representation of the LTP experimental procedure. (**B)** The fEPSP slope of baseline of LTP experiment in APP/PS1 and WT hippocampus. (**C)** Statistics of magnitude of LTP fEPSP slope for the first 10 min in APP/PS1 and WT hippocampus CA1. (**D)** Statistics of magnitude of LTP fEPSP slope for the last 10 min in APP/PS1 and WT hippocampus CA1. (**E)** Statistics of the location and length of AIS AnkG recorded after brain slices were induced LTP. Brain slices were induced LTP and fixed and stained with AnkG antibody. AIS plasticity of WT neurons was induced by LTP induction, while APP/PS1 neurons had no change in the AIS. (**F)** The fEPSP slope of baseline of LTP experiment in blebbistatin‐treated brain slices with control group. (**G)** Immunostaining results of glucose treatment in cultured neurons after being treated with blebbistatin. Red, AnkG; green, MAP2; blue, DAPI. White arrows indicate the ends of the AIS marked by AnkG staining. Scale bar, 20 µm. (**H)** Statistics of the location of the AIS marked by AnkG staining in blebbistatin‐treated neurons with the control group. The “Null” group stands for the neurons without blebbistatin and glucose treatment. The “Control” group stands for the neurons with blebbistatin treatment but no glucose treatment. Data were analyzed by one‐way analysis of variance (ANOVA). (H) Statistics of the length of the AIS marked by AnkG staining in blebbistatin‐treated neurons with the control group. Data were analyzed by one‐way analysis of variance (ANOVA). (**I)** Immunostaining results of cultured neurons after being treated with isoflurane and LTP. Red, AnkG; green, MAP2; blue, DAPI. White arrows indicate the ends of the AIS indicated by AnkG staining. Scale bar, 20 µm. (**J)** Statistics of the length of the AIS indicated by AnkG staining in isoflurane treated neurons after LTP with the control group. Data were analyzed by Student's *t*‐test.

LTP is a feature in the CNS that is related to be involved in learning and memory^34^ (Figure 6A). LTP experiments performed on isolated brain slices showed that the field excitatory postsynaptic potential (fEPSP) slope of APP/PS1 was significantly different from that of the control (Figure 6B‐D). LTP is closely related to neuronal plasticity, and the most critical one is synaptic plasticity.^35^, ^36^ Inspired by the disruption of plasticity, we considered that the deficits of AIS plasticity in APP/PS1 might be related to its abnormal LTP. We first evoked LTP and then detected alterations in its AIS position and length. We found that in WT mouse hippocampal slices, AIS plasticity was induced by the process that induced LTP (Figure 6E). However, in APP/PS1 hippocampal slices, the process of inducing LTP failed to produce plastic changes in the AIS (Figure 6E).

AIS plasticity mainly involves the movement of cytoskeleton proteins in the AIS. Blebbistatin, a myosin II inhibitor,^37^, ^38^ was used to inhibit this process (Figure 6G‐J). After inhibition, glucose treatment could no longer induce AIS plasticity, so we induced LTP of ex vivo brain slices under the premise of adding blebbistatin to the media, and the results showed that fEPSP slope showed significant changes in the treatment group (Figure 6F). Previous studies have showed that blebbistatin does not cause changes in the electrophysiological activity of neurons.^39^ The results implied that the plasticity of AIS might be necessary during theta burst‐induced LTP. The effects of using blebbistatin may extend beyond inhibiting the normal function of myosin II, yet we cannot ascertain this with certainty. To directly target the AIS and examine its impact using brain slices, we would have to utilize proteins that are specifically present at the AIS as targets for the intervention.

### 2.6 The giant 480 kDa AnkG could rescue electrophysiological deficits and impaired LTP in AnkG knockout mice

**FIGURE 7:**
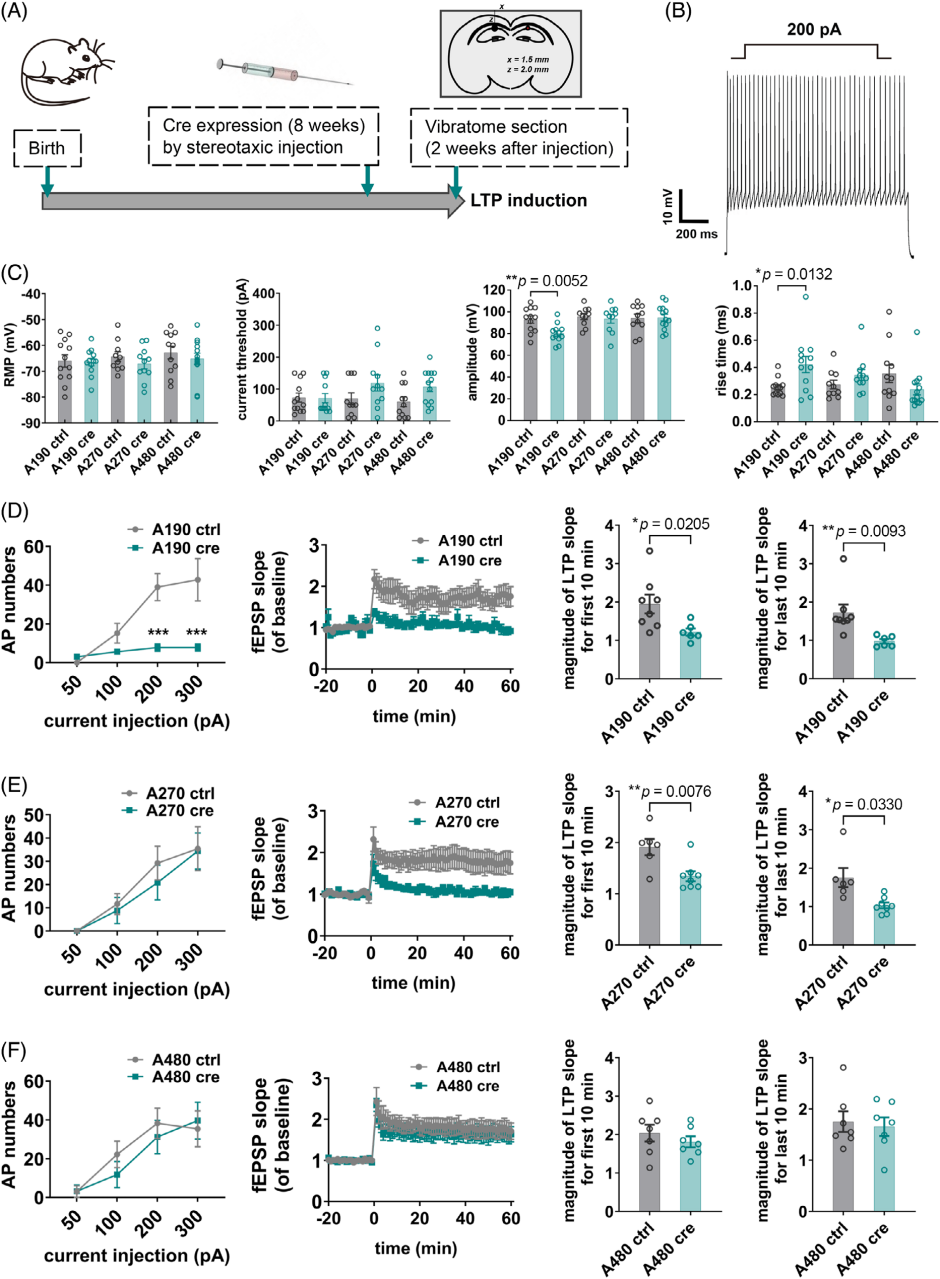
**Only 480 kDa AnkG isoform‐specific neurons and brain slices showed normal electrophysiological characteristics among all three kinds of single isoform AnkG expressing system. (A)** Schematic diagram of virus injection of AnkG related strains. Stereotaxic brain injection was performed on 2‐month‐old mice. Vibratome sectioning was performed and the corresponding slice was used for LTP induction. (**B)** An example of the cell membrane potential detected using a 200‐pA injected current. (**C)** Statistics of whole‐cell‐patch recording results. RMP, resting membrane potential. Data were analyzed by Student's *t*‐test. (**D)** The action potential number results of the whole cell patch experiment and fEPSP slopes of baseline of the LTP experiment in the A190 (190 kDa AnkG isoform specific) mice and control group. (**E)** The action potential number results of the whole cell patch experiment and fEPSP slopes of baseline of the LTP experiment in the A270 (270 kDa AnkG isoform specific) mice and control group. (**F)** The action potential number results of the whole cell patch experiment and fEPSP slopes of baseline of the LTP experiment in the A480 (480 kDa AnkG isoform specific) mice and control group. Data were analyzed by Student's *t*‐test.

Considering that A480 mice can restore AIS plasticity, we tried to induce LTP for all three‐isoform specific AnkG mice to observe the effects of different isoforms of AnkG on LTP. Cre expression was accomplished by stereotactic injection into the hippocampus at 8 weeks of age (Figure 7A). LTP induction was performed 2 weeks after injection. Patch‐clamp experiments showed that the basic electrophysiological parameters of the two larger isoform expressing mice were normal (Figure 7B), except those neurons containing only 190 kDa AnkG had fewer AP numbers, lower amplitude, and higher rise times (Figure 7C). LTP results showed that only A480 mice did not cause significant differences in fEPSP slopes compared with the control group, while the other two strains caused significant differences in fEPSP (Figure 7D–F). These results suggested that the presence of isoform 480 kDa could be critical for induction of LTP in the hippocampus and correspond to the finding that isoform 480 kDa AnkG compensates for AIS plasticity, which reinforces the understanding of the link between LTP and AIS plasticity. Notably, the A480 strain was able to restore the spatial memory and short‐term memory in behavioral tests, with the same trend as the LTP induction and AIS plasticity.^12^

## 3 DISCUSSION

Our study primarily focused on the early pathogenesis of AD, highlighting AIS shortening, altered electrophysiological properties, and impaired AIS plasticity during early stages of AD. We reported that these pathological changes in the AIS are correlated with functional abnormalities in AD model mice, such as a decline in LTP induction. By now, we still have limited understanding about early pathogenesis of AD. In recent years, the neuroinflammation theory related to microglia attracts more and more attention.^44^, ^45^ The activation of microglia often precedes obvious behavioral changes. It is reported that a part of microglia specifically affected the AIS from the developmental stage, and this effect no longer exists after the activation of microglia.^46^ Taken together with abnormal activation of microglia in AD, the changes in AIS during the early stages of AD may be related to the activation of microglia in this process. This may explain why the changes in AIS even precede obvious behavioral changes.

Multiple studies have indicated changes in the AIS in AD mouse models. In APP/TTA mouse model, the number of AIS decreases around Aβ plaques, accompanied by an increase in microglial cell density in the neighboring areas. In the same mouse model, AIS length shortens around Aβ plaques at 11 months of age, while no changes are observed at 6 months of age, which is in line with our research findings.^40^ These results suggested that the alterations of AIS length or position is associated with the burden of Aβ in the brain. Additionally, results have shown that directly adding APP to cultured cells can affect their AIS, causing it to shorten or move away from the cell body, which is more pronounced in neurons cultured with the APPswe mutation.^41^ In R1.40 AD mouse model, APP that transfected into neurons was detected in the AIS, which is not usually happened in the normal neurons.^41^ All the above results, along with our experiments, indicate that the AIS undergo pathological changes due to heritable Aβ‐related mutations, implying that changes in the AIS may play a role as downstream effects of Aβ overproduction. Apart from Aβ, tau pathology can also alter the plasticity and excitability of the AIS. The FTD‐related tau mutation V337M shortens the AIS and damages its plasticity in hiPSCs.^42^

In our study, we demonstrate that shortening of AIS in AD mouse models is related to reduction of neuronal excitability. Many previous studies have pointed out that the structural changes of the AIS are related to changes in neuronal excitability.^25^, ^27^, ^43^, ^44^ It is reported that rapid shortening of the AIS is directly associated with reduction of neuronal excitability with multiple AP firings.^45^ Researchers believed that the rapid plasticity mechanism of the AIS is related to the generation of LTP.^45^ A recent study has shown that damage to structural plasticity of the AIS is closely related to the change in excitability of ALS motor neurons, where AIS length of hiPSC MNs from early ALS increased and plasticity was impaired, which is directly related to its inherent hyperexcitability of neurons.^28^ They found that increase in AIS length is related to decrease in the rheobase of its neurons, consistent with our findings in AD mouse model neurons that the AIS is shortened and the rheobase is increased. This fully illustrates that length change of the AIS and its altered plasticity are closely related to the electrophysiological properties of neurons.

Our current study still has limitations. In the future, it is necessary to further explore the mechanism of AIS plasticity, and the impact of AIS plasticity on neuronal excitability. Especially, the mechanism of how AIS plasticity is associated with LTP induction is still unclear. On the other hand, a more multi‐level variable of time points should be introduced when studying the changes in AIS and the expression changes of AnkG to ensure detailed understanding of early changes during AD development.

Overall, we have studied the changes in the length and electrophysiological activity of AIS in the early pathological progression of AD, and on this basis, we have observed the impairment of plasticity in AIS in AD. Our observations support that the AnkG 480 kDa isoform is crucial for the maintenance of AIS plasticity. These results are of some significance for understanding the pathological changes of neurons in the early pathological progression of AD and the role of AIS in the progression of AD.

## 4 MATERIALS AND METHODS

### 4.1 Human samples

Brain tissues for western blotting and immunohistochemistry were sourced from the Brain Bank and the Neurodegenerative Disorder Research Centre at the University of Science and Technology of China (USTC). The procedure for preserving postmortem adult human brain tissues involved two primary steps: initially, the unfixed brain tissue was rapidly frozen, a method known as snap‐freezing; subsequently, the tissue underwent chemical fixation followed by storage at a temperature of −80°C within the human brain bank.

### 4.2 Animals

We bought APP/PS1 mice from Shanghai Model Organisms Center, and 5xFAD and 3xTg from Beijing Jitianbio Biotechnology Co. LTD. AnkG^fl/fl^ mice were kindly provided by Dr. Vann Bennett (Duke University).^33^ The establishment of AnkG 190/270/480‐TG mice was finished at the Shanghai Model Organisms Center.^12^ Additionally, the creation of AnkG 190/270‐TG mice involved the use of CRISPR/Cas9 technology for targeted insertion of the CAG‐loxP‐3×stop‐loxP‐190‐kDa AnkG‐IRES‐EYFP‐WPRE‐polyA or CAG‐loxP‐3×stop‐loxP‐270‐kDa AnkG‐IRES‐RFP‐WPRE‐polyA sequence at the Rosa 26 locus on Chromosome 6. For the production of AnkG 480‐TG mice, the PiggyBac plasmid containing CAG‐loxP3 polyA‐loxP‐480‐kDa AnkG‐IRES‐EGFP‐WPRE‐polyA was introduced into oosperm using the PiggyBac transposase system.

### 4.3 Antibodies

The primary antibodies used were as follows: anti‐AnkG (1:2000 for western blot and 1:1000 for immunostaining, OM167212; OmnimAbs), mouse anti‐βIV‐spectrin (1:500, N393/76; NeuroMab), mouse anti‐Nav1.2 (1:500, K69/3; NeuroMab), mouse anti‐neurofascin (1:500, A12/18; NeuroMab), and chicken anti‐MAP2 (1:10000, ab5392; Abcam). The secondary antibodies used for immunostaining were from Invitrogen AlexaFluor series. The HRP‐conjugated antibodies used were from Easybio, diluted as 1:2000.

### 4.4 Western blots

When dissecting out the hippocampus, 200 µL of RIPA lysis buffer (Beyotime, P0013B) with protease inhibitor (Roche, cOmplete cocktail, 1 tablet per 10 mL lysis buffer) was added. After addition of lysis buffer, the tissue was fully ground on ice for 10 min. After grinding, the samples were refrigerated for 10 min, vortexed for 5 s, and then returned to the refrigerator for another 10 min. This vortex and refrigeration cycle was repeated three times. The suspension was centrifuged at 16,000 × *g* for 20 min at 4°C and then the supernatant was obtained. Proteins were denatured using 4× LDS buffer (Invitrogen, NP0007) and heated at 70°C for 10 min. Protein concentration was measured by Pierce BCA Protein Assay Kits (ThermoFisher Scientific, 23227). Use 3%−8% tris‐acetate gel (ThermoFisher Scientific, EA0378BOX) to operate gel electrophoresis. Note that 10 µL of 20 µg protein was loaded in each well. The gel was run at 150 V for 55 min, and 0.45‐µm PVDF membrane was used for transfer and transfer buffer (ThermoFisher Scientific, NP0006) was diluted with two times methanol and 17 times water. Before using the membrane, it was soaked in pure methanol for 30−45 s. The transfer was initiated by a constant voltage of 25 V and lasted for 72 min. Blocking solution contained 5% skim milk, prepared with tris buffered saline with Tween‐20 (TBST, and incubated at room temperature for 1 h on a shaker. Following the blocking step, the primary antibody was incubated by dissolving in the blocking solution, and the membrane was incubated overnight at 4°C on a shaker. The secondary antibody was mixed at the ratio of 1:2000 in the blocking solution, and membrane was incubated at room temperature for 1 h on a shaker. ThermoFisher Scientific SuperSignal West Dura (Cat. 34075) was used for enhanced chemiluminescence (ECL) chemiluminescent analysis.

### 4.5 Immunocytochemistry

The cultured cells fixed by 4% paraformaldehyde (PFA) at room temperature for 20 min, permeabilization with phosphate buffered saline with Tween‐20 (PBST) with 0.2% Triton‐X at 4°C for 15 min, and blocking with PBST solution with 5% bovine serum albumin (BSA) in it at room temperature for 1 h. PBST is made by adding 0.1% tween into phosphate buffered saline (PBS). Primary antibody is added and incubated at 4°C overnight. Secondary antibody is added at the ratio of 1:500 at room temperature for 1 h, avoiding light. 4',6‐diamidino‐2‐phenylindole (DAPI) staining is performed for nuclei at room temperature for 10 min. Fluoromount‐G is for slides mounted.

### 4.6 Immunohistochemistry

Take adult mice and use abdominal injection to administer anesthesia. For C57 mice, we used 300 µL of 1% pentobarbital. The mouse was kept in a dark and quiet environment for 5 min until it was completely anesthetized. Note that 40 mL of sterile PBS was injected to replace the mouse's blood. Twenty milliliters of 4% PFA was injected into the mouse's whole‐body circulation to quickly fix the mouse's tissues. The mouse brain was placed in 4% PFA and fixed at 4°C for 4 h. The fixed mouse brain was placed in PBS with 20% sucrose at 4°C for 12 h, and then placed in PBS with 30% sucrose at 4°C for 12 h. The brain was placed in a small box with optimal cutting temperature compound (O.C.T.). After sectioning, PBST solution containing 0.3% Triton X‐100 was added and the brain slices were incubated for 15 min. The PBST solution containing 5% BSA was added and incubated for 2 h to block. It was incubated with primary antibody overnight, and secondary antibody was added for 2 h at room temperature. It was stained with DAPI (500 µL per well) at room temperature for 15 min.

### 4.7 Electrophysiology

Whole‐cell patch‐clamp recordings were conducted on hippocampal neurons. Patch pipettes utilized for these somatic recordings, when filled with an internal solution comprising (in mM) 140 potassium gluconate, 3 KCl, 2 MgCl_2_, 2 Na_2_ATP, 0.3 Na_3_GTP, 10 HEPES, and 0.2 EGTA (adjusted to pH 7.2 with KOH), exhibited resistances ranging from 3 to 6 MΩ. Throughout the recording process, the access resistance was regularly monitored and compensated up to a maximum of 70%. Somatic recordings exhibiting an access resistance exceeding 25 MΩ were excluded. Adjustments for bridge balance and capacitance compensation were meticulously performed at the start and end of each experimental procedure.

For extracellular field potential recordings in this study, a commercially available 64‐channel multisite recording system (MED64, Panasonic Alpha‐Med Sciences) was employed. The preparation protocol for the MED followed the methodology previously described.^46^ Each electrode within the array measured 50 × 50 µm, and the 64 electrodes were arranged in an 8 × 8 grid with an inter‐electrode spacing of 300 µm, covering a total area of 4.4 mm^2^. The surface of the MED64 probe was coated overnight at 4°C with 0.01% poly‐l‐ornithine before use (Sigma, P4957). Then the probe surface was washed thrice with sterile distilled water for immediate use in experiments. The current sources and sinks from all 64 electrodes were transformed into two‐dimensional current source density images using bilinear interpolation at each point. After incubation, a brain slice was placed onto the pre‐treated MED64 probe and perfused with oxygenated artificial CSF (95% O_2_ and 5% CO_2_) at a flow rate of 2 mL/min and maintained at a temperature of 28−30°C. Current pulse stimulation (1–10 mA, 0.2 ms) was administered to the stimulation channel, adjusting the intensity to elicit a half‐maximal field fEPSP in the channels proximal to the stimulation site. Channels that displayed fEPSPs were deemed active, and their fEPSP responses were sampled every minute. The maximum fEPSP from the SC pathway was selected for analysis in each slice.^47^ The term “slope” refers to the mean slope of each fEPSP recorded in the active channels. A stable baseline response was initially established, ensuring that the variation in baseline response was less than 5% within 30 min for most active channels.

### 4.8 Stereotactic injection

Mice first underwent anesthesia as it s described in the Immunohistochemistry method part, followed by the hippocampal injection (stereotactic coordinates: 2.1 mm posterior to the bregma; 1.8 mm lateral to either side of the midline; 2.8 mm ventral from the top of the skull) of 1 µL of either pAAV‐hSyn‐cre‐p2A‐bfp or pAAV‐hSyn‐p2A‐bfp (titer 10^12^) using a microinjector linked to a microinjection pump and attached to a stereotaxic apparatus (RWD Life Science) for AAV vector transfection. The mice were then kept on a heating blanket at 37°C until they fully awoke from anesthesia. Mice were given bilateral injections when they were 2 months old and behavioral testing and brain slice sectioning were performed 2 weeks later.

### 4.9 Primary neuronal culture

Neurons cultured in vitro for imaging were maintained on glass slides. Each slide was coated with approximately 200 µL of poly‐d‐lysine (Sigma‐Aldrich) and incubate the slide for over 60 min. A pair of hippocampi from mice was removed by dissection and placed in DMEM on ice. To operate digestion of the hippocampus, the DMEM was removed from the dish and 2 mL of trypsin (*t*_0.25_, 25200056; Invitrogen) was added and incubate for 20 min at 37°C. Two milliliters of DMEM/F12 medium supplemented with 10% fetal bovine serum (DF12) was used to halt the digestion process and resuspend the cells. After settling for 2 min, the supernatant was obtained and centrifuged at 500 × *g* for 2 min. The supernatant was discarded and the cell was resuspended in 900 µL of DF12 per pair of hippocampi. Note that 200 µL of the cell suspension was added to each well. The plate was incubated in a cell culture incubator for over 30 min. Subsequently, 2 mL of complete neuronal basal medium (CNB) was added to each well. For media changes, approximately 1 mL of media from each well was aspirated and replaced with an equal volume of fresh CNB.

### 4.10 Neuron modeling and data processing

The python (3.9) version of NEURON (8.2.4) was used for modeling the AP presented on the AIS. The neuron model is divided into four sections (the soma, dendrites, AIS and axon). The complete analysis code can refer to https://github.com/Wistomize/neuronmodel. The 3D plots were plotted using matplotlib (3.8.0). The length and the location of the AIS were measured with ImageJ. The segmented line tool and plot profile tool of ImageJ are used for this purpose. The AIS is defined as a continuous segment where the fluorescence intensity of AnkG is greater than one‐third of the peak brightness of that segment. Both the location and length of the AIS are determined exactly based on this definition.

### 4.11 Statistics

GraphPad Prism 10.0 was used to perform statistical analyses. Data are presented as means ± SEM. Statistical significance between groups with normally distributed data was calculated by Student's *t*‐test or one‐way analysis of variance followed by Dunnett's multiple comparisons test. *p* < 0.05 was considered significant: ∗*p* < 0.05, ∗∗*p* < 0.01, ∗∗∗*p* < 0.001, or ∗∗∗∗*p* < 0.0001. More details can be found in the figure legends.

## AUTHOR CONTRIBUTIONS

Y.L., H.W., Y.W., Z.C., Y.L., and W.T. performed all experiments and analyzed the data. X.K., A.P., D.K., and H.W.L. helped in modeling, cellular assays, animal behavioral tests, and neuronal culture. X.Y. helped in processing of human samples and analyzing the data. Y.Z. conceptualized the study, performed analyses, and drafted the manuscript with inputs from all authors. All authors have read and approved the final manuscript.

## CONFLICT OF INTEREST STATEMENT

All authors declare no actual or potential conflicts of interest, including any financial, personal or other relationships, with other people or organizations within 3 years of beginning the work submitted that could inappropriately influence (bias) their work.

## ETHICS STATEMENT

All animal studies were conducted in accordance with the Animal Care and Use Guidelines of Peking University, with approval number LSC‐ZhangY‐1.

## Supporting information



Supporting Information

## Data Availability

No sequence data were used in this study.
