# Supplementary material for: Alterations in the axon initial segment plasticity is involved in early pathogenesis in Alzheimer's disease
Source: MedComm (2020). 2024 Oct 14;5(11):e768. doi: 10.1002/mco2.768 (PMC11473794; doi:10.1002/mco2.768)
Supplement: Supplementary file 1 — Supporting Information [file MCO2-5-e768-s001.pdf]

Supporting Information of

# Alterations in the axon initial segment plasticity is involved in early pathogenesis in Alzheimer's disease

## Supplementary Figure 1.

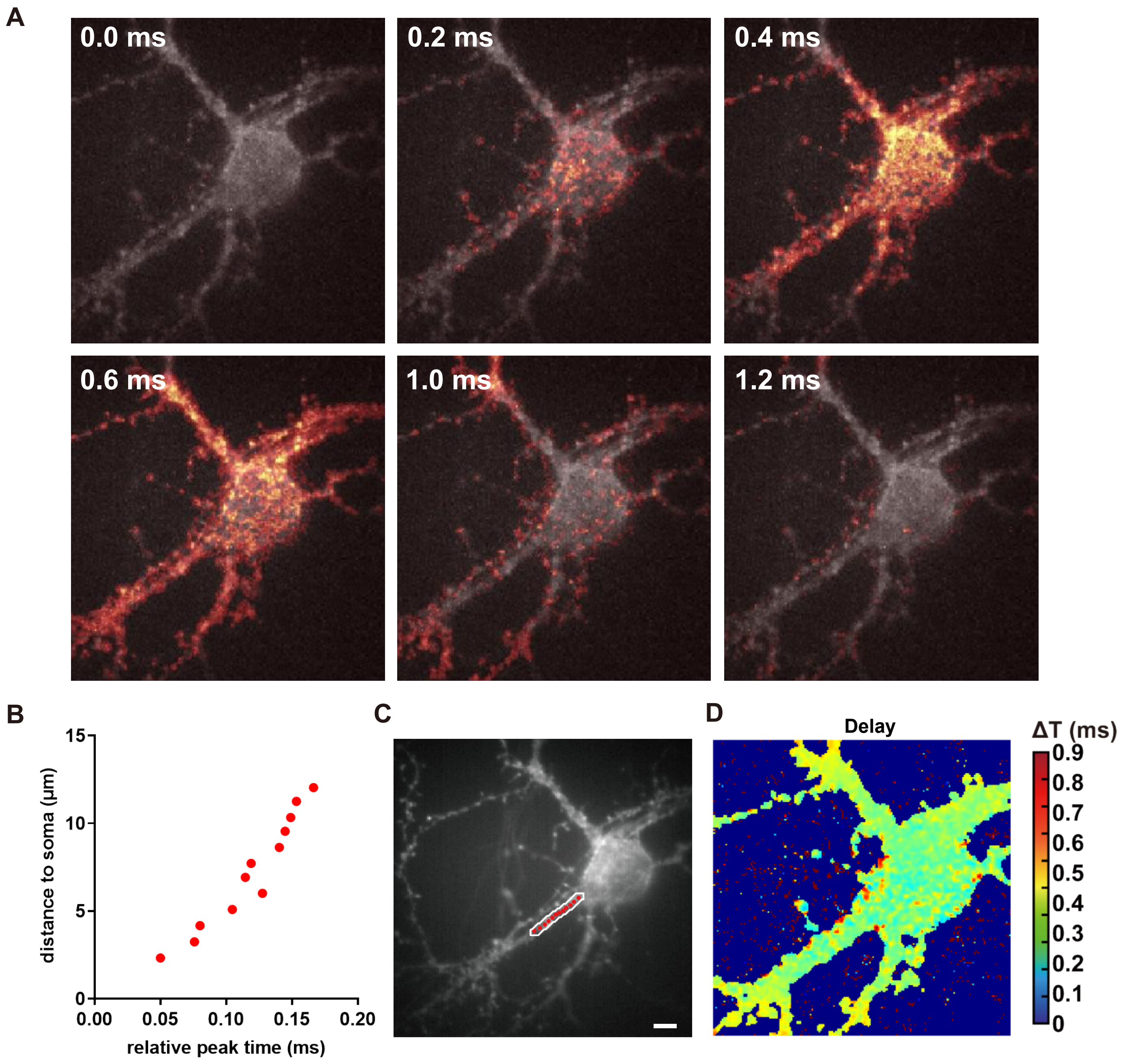


**Figure S1. Back propagation action potentials on dendrites were observed using a membrane potential probe.**

**A**. Images of membrane potential probe fluorescence after current injection into a neuron. **B**. The obtained distance to the soma was calculated as a function of the relative peak time using the fluorescence imaging data. **C**. Location of the AIS at the mark in the black and white fluorescence image (red line segment). **D**. Delays and spatial distribution of membrane potential changes following an action potential burst.

# Supplementary Figure 2.

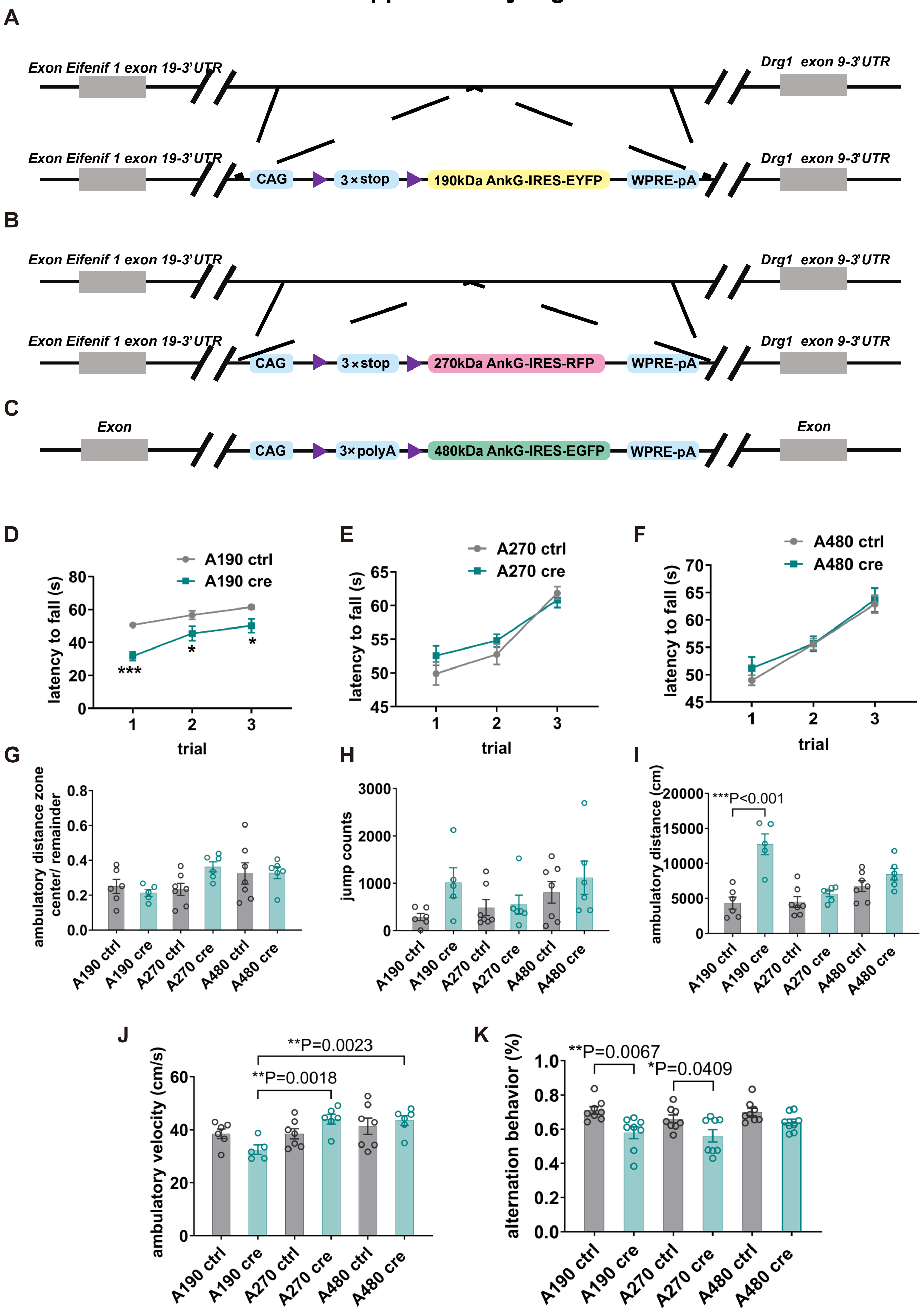

**Figure S2. Construction schematic diagrams of the three AnkG KI mouse strains and behavioral results of the three single isoform AnkG expressing mice.**

**A**.**B**.**C**. Diagram of knock-in gene patterns for the three AnkG isoforms. (**A**.) 190 kD AnkG KI, (**B**.) 270 kD AnkG KI and (**C**.) 480 kD AnkG KI. The three knock-in models carried different fluorescence with IRES label. **D**.**E**.**F**. The latency to fall in second of the results of the rotarod tests. The rotarod tests were performed with the three AnkG single isoform expressing mice and the control group. **G**. The ambulatory distance in the center zone vs. the remainder zone in the OFT (open field test) experiments. The experiments were performed with the three AnkG single isoform expressing mice and the control group. **H**. The counts of jump of the results of the OFT tests. The tests were performed with the three AnkG single isoform expressing mice and the control group. **I**. The ambulatory distances (cm) of the results of the OFT tests. **J**. The ambulatory velocity (cm/s) of the results of the OFT tests. **K**. The alternation behavior of the results of the Y-maze tests. The Y-maze tests were performed with the three AnkG single isoform expressing mice and the control group.

## Supplementary Figure 3.

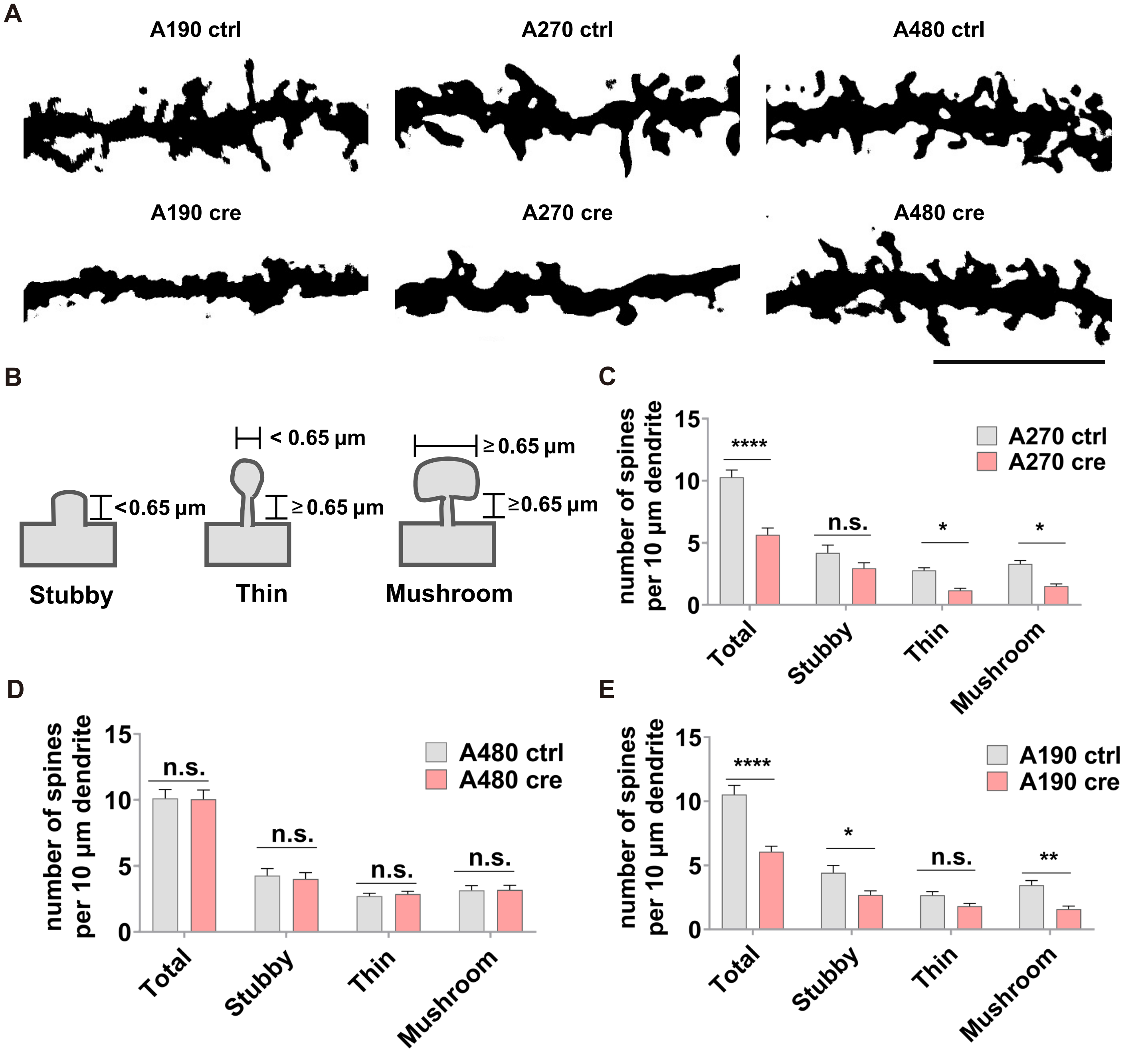


**Figure S3. Only 480 kD AnkG isoform expressing neurons have normal morphology and amounts of spines among the three kinds of AnkG isoform specific neurons.**

**A**. Sparsely-labeled dendritic spines of the three kinds of single isoform AnkG expressing mice with control group using Golgi staining. **B**. A schematic diagram of spine classification. Using deep learning image recognition, the stained spines were divided into "Stubby", "Thin" and "Mushroom" according to the morphology. **C**. The number of spines classified by the morphology per 10 μm dendrite of the A270 mice with the control group. **D**. The number of spines classified by the morphology per 10 μm dendrite of A480 mice with the control group. **E**. The number of spines classified by the morphology per 10 μm dendrite of A190 mice with the control group.

# Supplementary Figure 4.

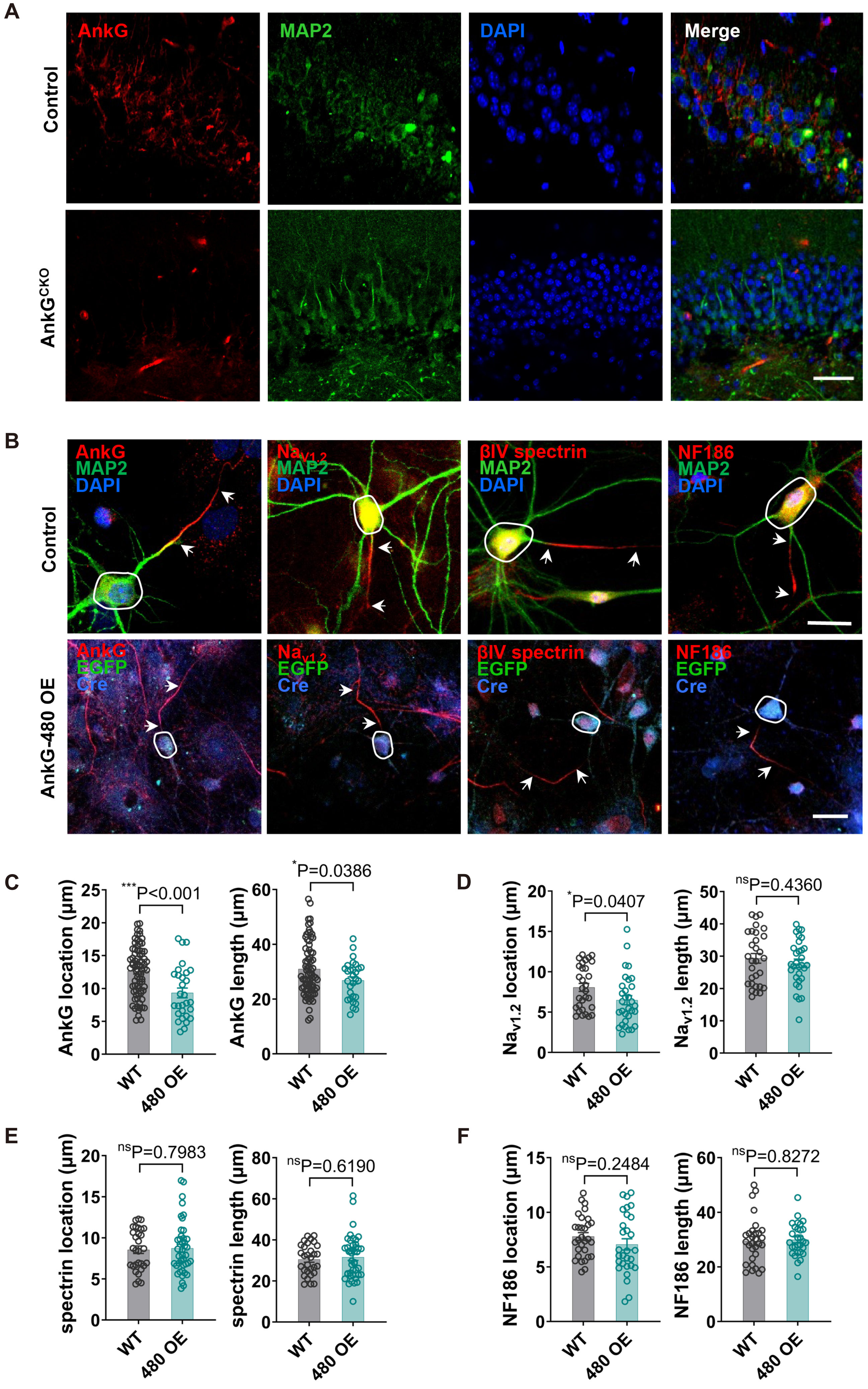

**Figure S4. Length and distance to the soma of AIS from 480 kD AnkG KI mice and AIS images of AnkG knockout mice.**

**A**. Fluorescence image of AnkG conditional knockout hippocampus. Red, AnkG (AIS), green, MAP2 (soma and dendrites), blue, DAPI (nuclei). Scale bar, 50 μm. **B**. Fluorescence image of cultured neurons from 480 kD AnkG KI mice. Red, AIS markers (AnkG, $Na_{V1.2}$, βIV spectrin, NF186), green, EGFP, blue, Cre-BFP. Scale bar, 50 μm. **C**. The AIS location (left) and length (right) of cultured neurons in 480 kD AnkG KI mice marked by AnkG with control group. **D**. The AIS location (left) and length (right) of cultured neurons in 480 kD AnkG KI mice marked by $Na_{V1.2}$ with control group. **E**. The AIS location (left) and length (right) of cultured neurons in 480 kD AnkG KI mice marked by βIV spectrin with control group. **F**. The AIS location (left) and length (right) of cultured neurons in 480 kD AnkG KI mice marked by NF186 with control group. Data were analyzed by Student's t-test.

**Supplementary Figure 5.**

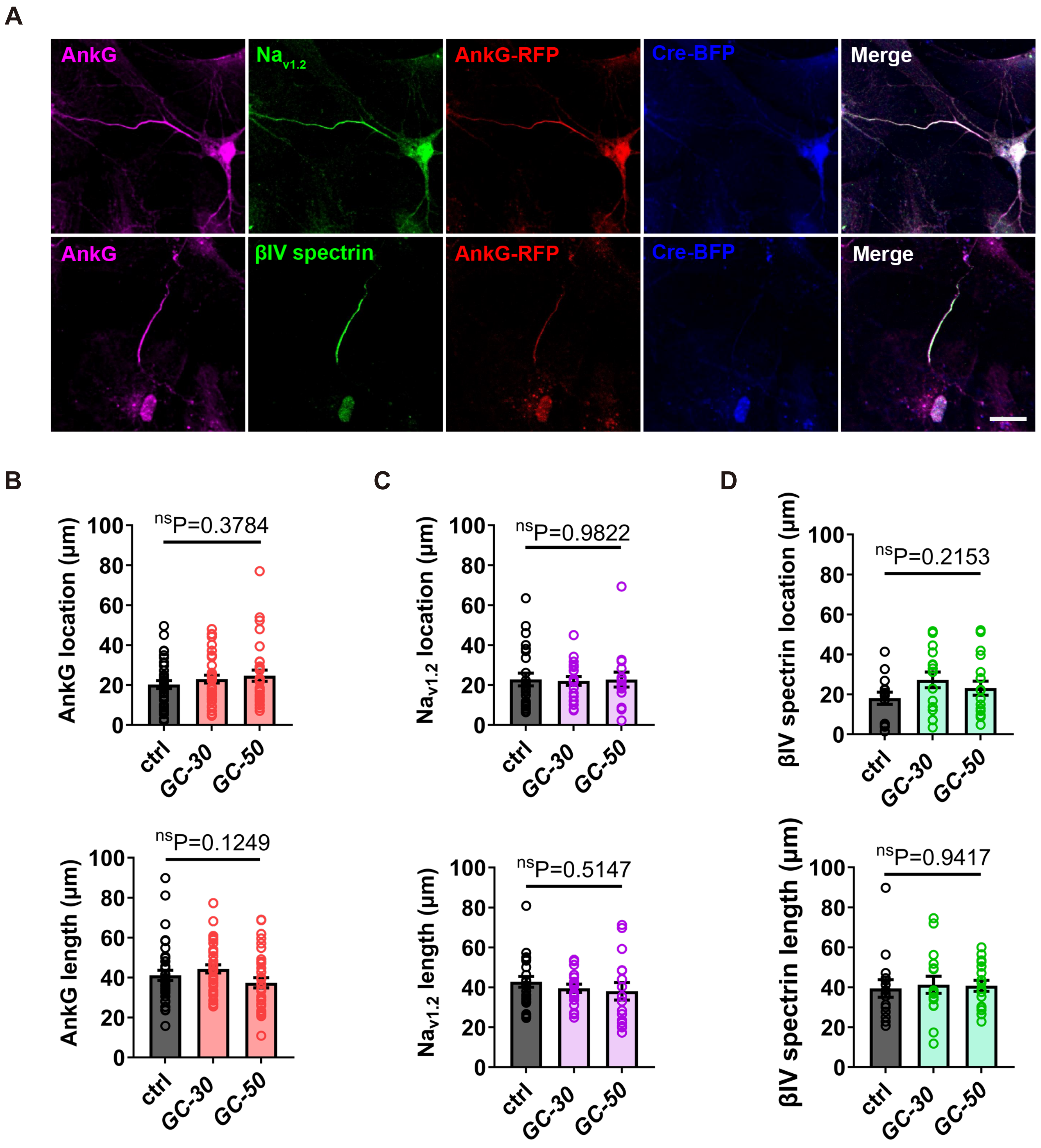

**Figure S5. Length and distance to the soma of AIS from 480 kD AnkG KI mice and AIS images of AnkG knockout mice.**

**A**. Neurons from 270 kD AnkG isoform specific mice (A270) were treated with 30 mM glucose on DIV 3. And neurons were fixed and stained on DIV 7. Scale bar, 40 μm. **B.** Statistics of the location and length of AnkG from A270 neurons after being treated with 10 mM, 30 mM or 50 mM glucose for 10 h. **C.** Statistics of the location and length of $Na_{v1.2}$ from A270 neurons after being treated with 10 mM, 30 mM or 50 mM glucose for 10 h. **D.** Statistics of the location and length of βIV spectrin from A270 neurons after being treated with 10 mM, 30 mM or 50 mM glucose for 10 h. Data were analyzed with one-way ANOVA. Significance level was set as 0.05.
